# Genome-Wide Analyses of the *XTH* Gene Family in *Brachypodium distachyon* and Functional Analyses of the Role of *BdXTH27* in Root Elongation

**DOI:** 10.3390/ijms26157457

**Published:** 2025-08-01

**Authors:** Hongyan Shen, Qiuping Tan, Wenzhe Zhao, Mengdan Zhang, Cunhao Qin, Zhaobing Liu, Xinsheng Wang, Sendi An, Hailong An, Hongyu Wu

**Affiliations:** 1 College of Horticulture Science and Engineering, Shandong Agricultural University, Tai’an 271018, China; 17863808135@163.com (H.S.); dundunhappy@126.com (Q.T.); 2022110301@sdau.edu.cn (W.Z.); 2 National Center of Technology Innovation for Saline-Alkali Tolerant Rice in Sanya, Sanya 572000, China; 3 College of Life Sciences, Gannan Normal University, Ganzhou 341000, China; 4 State Forestry Administration Key Laboratory of Silviculture in Downstream Areas of the Yellow River, College of Forestry, Shandong Agricultural University, Tai’an 271018, China; 17866927099@163.com (M.Z.); wxs13173051098@163.com (X.W.); ansendi@163.com (S.A.); 5 National Key Laboratory of Wheat Breeding, College of Life Sciences, Shandong Agricultural University, Tai’an 271018, China; qincunhao@163.com (C.Q.); 17866708179@163.com (Z.L.)

**Keywords:** *Brachypodium distachyon*, xyloglucan endotransglucosylase/hydrolase, genome-wide analysis, gene expression

## Abstract

Xyloglucan endotransglucosylase/hydrolases (XTHs) are a class of cell wall-associated enzymes involved in the construction and remodeling of cellulose/xyloglucan crosslinks. However, knowledge of this gene family in the model monocot *Brachypodium distachyon* is limited. A total of 29 *BdXTH* genes were identified from the whole genome, and these were further divided into three subgroups (Group I/II, Group III, and the Ancestral Group) through evolutionary analysis. Gene structure and protein motif analyses indicate that closely clustered *BdXTH* genes are relatively conserved within each group. A highly conserved amino acid domain (DEIDFEFLG) responsible for catalytic activity was identified in all BdXTH proteins. We detected three pairs of segmentally duplicated *BdXTH* genes and five groups of tandemly duplicated *BdXTH* genes, which played vital roles in the expansion of the *BdXTH* gene family. *Cis*-elements related to hormones, growth, and abiotic stress responses were identified in the promoters of each *BdXTH* gene, and when roots were treated with two abiotic stresses (salinity and drought) and four plant hormones (IAA, auxin; GA3, gibberellin; ABA, abscisic acid; and BR, brassinolide), the expression levels of many *BdXTH* genes changed significantly. Transcriptional analyses of the *BdXTH* genes in 38 tissue samples from the publicly available RNA-seq data indicated that most *BdXTH* genes have distinct expression patterns in different tissues and at different growth stages. Overexpressing the *BdXTH27* gene in *Brachypodium* led to reduced root length in transgenic plants, which exhibited higher cellulose levels but lower hemicellulose levels compared to wild-type plants. Our results provide valuable information for further elucidation of the biological functions of *BdXTH* genes in the model grass *B. distachyon*.

## 1. Background

Xyloglucan endotransglucosylase/hydrolases (XTHs), a subfamily of the glycoside hydrolase family GH16, are crucial enzymes that are involved in the regulation of cell wall extension, construction, and degradation in plants [1,2]. XTH proteins have two significant catalytic activities, and can act either as endotransglucosylases (XET, EC 2.4.1.207) to elongate xyloglucan chains by cleaving the chains and rejoining the reducing ends to other xyloglucan molecules, or as endohydrolases (XEH, EC 3.2.1.151) that cleave xyloglucan chains by rejoining the xyloglucan reducing end to water molecules [3]. XTH proteins are predicted to present several common structural features: a putative signal peptide, a conserved ExDxE motif likely to be the catalytic site for both XET and XEH activities, a potential *N*-glycosylation site necessary for protein stability, and several cysteine residues that stabilize the C-terminal end [4].

The *XTH* gene family is widely distributed in both monocotyledonous and dicotyledonous plants, and the gene numbers vary within individual plant species, with 33 in Arabidopsis [5], 29 in rice (*Oryza sativa*) [6], 25 in tomato (*Solanum lycopersicum*) [7], 135 in wheat [8], 38 in *Osmanthus fragrans* [9], 30 in sugar beet [10], 29 in Chinese jujube (*Ziziphus jujuba*) [11], 38 in poplar (*Populus cathayana*), 32 in willow (*Salix rehderiana*) [12], 19 in *Boehmeria nivea* [13], 22 in mulberry (*Morus alba* L.) [14], 53 in banana (*Musa* spp.) [15], 53 in *Brassica rapa*, and 38 in *Brassica oleracea* [16]. The XTH proteins were clustered into three groups (I, II, and III) on the basis of sequence similarity in *A. thaliana* [4,5]. In rice, the XTH proteins were found to cluster into two major groups named I/II and III because the boundary between Groups I and II was not apparent [6]. Baumann et al. (2007) [17] used ~130 full-length XTH protein sequences mainly from *Arabidopsis*, rice, black cottonwood (*Populus trichocarpa*), tomato, and hybrid aspen (*Populus tremula* × *Populus tremuloides*) to derive a tree from a structure-based sequence alignment using the maximum likelihood method. This study showed that Group III can be further subdivided into two main clades (Group III-A and Group III-B), and a small outlying ancestral group that is close to the root. Many studies recently adopted a similar classification system for the *XTH* gene family, grouping members into Group I/II, Group III, and the Ancestral Group [8,9,11,12]. Furthermore, XTH proteins in Groups I, II, and III-B have been reported to have significant xyloglucan endotransglucosylase (XET) activity, while proteins in Group III-A mainly showed xyloglucan endohydrolase (XEH) activity [4,18,19].

On the one hand, XTH family genes are involved in many physiological responses. For example, *DkXTH1*, *DkXTH4*, and *DkXTH5* in persimmon show higher expression levels and are associated with fruit firmness. However, the expression levels of *DkXTH2* and *DkXTH3* reach their maxima concomitant with pronounced fruit softening [20]. In addition, overexpression of *FvXTH9* and *FvXTH6* might promote strawberry fruit ripening by modifying cell wall components [21]. *XTH17* and *XTH24* in *Arabidopsis* are involved in polar cell elongation [22]. The natural variation in *PtoXET16A* in poplar can affect wood properties, and the expression of *PtoXET16A* in *Populus tomentosa* was highest in the root, followed by the phloem, cambium, and developing xylem, suggesting that *PtoXET16A* plays important roles in the development of vascular tissues [23]. On the other hand, XTH family genes also play important roles in the response to plant hormones and abiotic stresses. For example, increasing the expression of the wheat *TaXTH17* gene in *Arabidopsis* leads to decreased drought tolerance. In contrast, silencing the *TaXTH17* gene in wheat through barley stripe mosaic virus (BSMV)-mediated gene silencing improves drought resistance [8]. The expression of Arabidopsis *XTH17* was substantially reduced in the presence of aluminum (Al), and the *xth17* and *xth31* mutants were more Al resistant than was the wild type [24]. Expression of *DkXTH6* in persimmon (*Diospyros kaki*) was found to be positively up-regulated during ethylene production, as well as by propylene and ABA treatments, although expression was down-regulated by GA3 and cold treatments. However, the mRNA levels of *DkXTH7* were the highest in GA3-treated fruits and cold-treated fruits [25]. In addition, overexpression of persimmon *DkXTH1* enhanced tolerance to salt, ABA, and drought stresses in transgenic Arabidopsis plants and delayed fruit softening in transgenic tomatoes [26].

*Brachypodium distachyon* is a species of monocot that is used as a model system for genetic and physiological studies in grasses. Plants of *B. distachyon* are small in stature, have a short life cycle, a small genome, modest growth requirements, and many available mutant resources [27,28,29,30,31]. Although *XTH* genes have been reported to play important roles during plant growth and development, the temporal and spatial expression patterns and specific biological functions of *BdXTH* genes in *Brachypodium* are largely unknown. The available genome sequence of *Brachypodium distachyon* and related studies of the *XTH* gene families from other species will enable a comprehensive characterization of *BdXTH* genes from *B. distachyon* [32,33]. In this study, we identified 29 *BdXTH* genes in the *B. distachyon* genome based on a bioinformatic analysis. We then performed a comprehensive analysis of the *BdXTH* genes, including their evolutionary relationships, gene structures, duplication events, conserved motifs, and *cis*-regulatory elements. To provide useful information for further functional studies of the *BdXTH* genes in *B. distachyon*, the expression patterns of the *BdXTH* genes in different tissues, at different developmental stages, and in response to stresses such as plant hormones, salinity, and drought were analyzed. Additionally, we conducted a preliminary analysis to evaluate the functional role of the *BdXTH27* gene. The results of this study provide necessary resources for further research into the specific functions and regulatory mechanisms of *BdXTH* genes.

## 2. Results

### 2.1. Identification of the BdXTH Genes in Brachypodium distachyon

**Figure 1:**
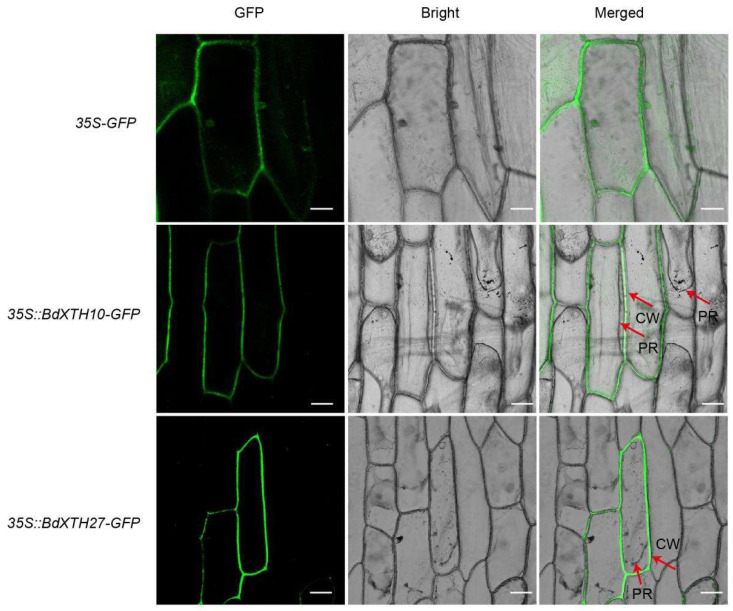
Subcellular localization of the BdXTH10 and BdXTH27 proteins. The control (*35S*-*GFP*) and fusion vectors (*35S*::*BdXTH10*-*GFP* and *35S*::*BdXTH27*-*GFP*) were transiently expressed separately in onion epidermal cells using agrobacterium-mediated transfection. CW, cell wall; PR, protoplast. scale bar = 50 μm.

The availability of the *B. distachyon* genome makes it possible to identify the *XTH* family genes on a genome-wide level. A total of 29 candidate *BdXTH* genes that are predicted to encode proteins containing both the PF00722 and PF06955 domains were identified. These genes were renamed *BdXTH1* to *BdXTH29* on the basis of their chromosomal positions (Table 1). The lengths of the predicted BdXTH proteins varied from 279 to 372 amino acids, with an average length of 306 amino acids. Corresponding with protein length, the molecular weights (MWs) ranged from 30.54 kDa to 40.94 kD. Subcellular localization analysis predicted that 27 BdXTH proteins are located in the cell wall, and the other two proteins, BdXTH8 and BdXTH23, are targeted to both the cell wall and the cytoplasm. In order to further verify the reliability of the predictions, we constructed an expression vector in which the *BdXTH10* and *BdXTH27* genes were fused in-frame with *GFP* and transiently expressed the fusion protein in onion epidermal cells. The result of this experiment shows that BdXTH10 and BdXTH27 are targeted to the cell wall (Figure 1). The predicted isoelectric points (PIs) of the BdXTH proteins range from 4.67 to 8.83 due to variations in their amino acid sequences. Information relating to other parameters of the BdXTH proteins, such as instability index (II), aliphatic index (AI), and grand average of hydropathicity (GRAVY), is also presented in Table 1.

### 2.2. Evolutionary Analysis of BdXTH Proteins

**Figure 2:**
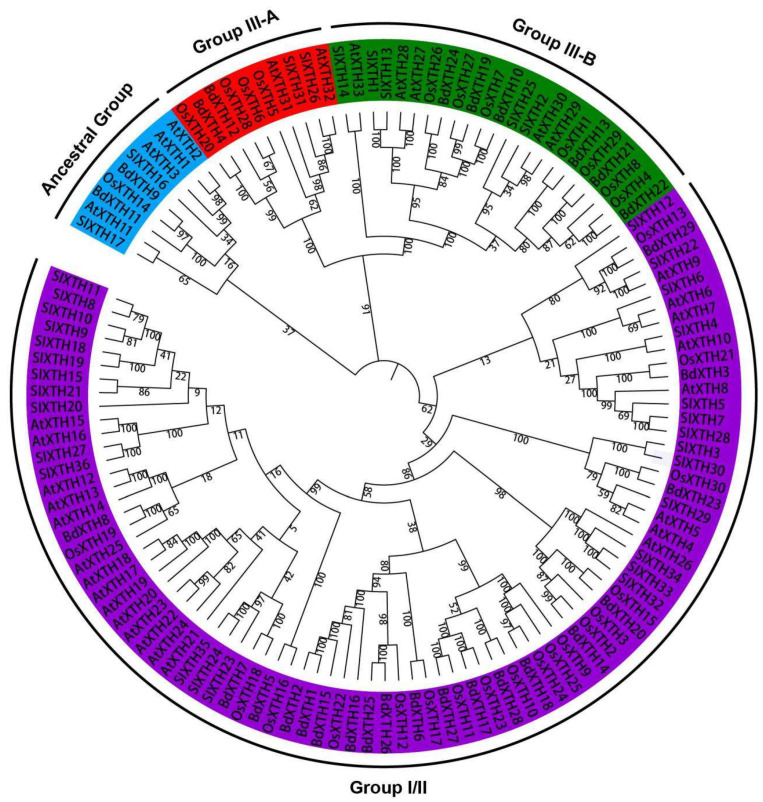
Evolutionary relationships among XTH proteins from *Brachypodium* and three other plant species. The tree was constructed with the Neighbor Joining (NJ) method as implemented in MEGA-X v10.1 software, and branch confidence was estimated by bootstrapping with 1000 replicates. The XTH proteins are classified into three major clades (Group I/II, Group III, and the Ancestral Group). Proteins in Group I/II and the Ancestral Group are shown with purple and blue backgrounds, respectively. Group III is further divided into two subclades, Group III-A and Group III-B, which are indicated with red and green backgrounds, respectively.

To study the evolutionary relationships among XTH proteins in dicots and monocots, a tree was constructed using the full-length candidate XTH protein sequences in *Brachypodium* and three other species, including 33 AtXTHs from *Arabidopsis*, 30 OsXTHs from rice, and 36 SlXTHs from tomato (Table S1). The results showed that the XTH proteins cluster into three main groups (Group I/II, Group III, and Ancestral Group), that each group contains XTH proteins from the four species (Figure 2), which suggests that the precursor genes were present in the most recent common ancestor of monocots and dicots, and that closely related proteins might perform similar functions in the different species. Not unexpectedly, proteins from closely related species clustered together. Proteins from the monocots (*B. distachyon* and rice) tended to cluster together, and proteins from the dicots (tomato and *Arabidopsis*) clustered together. Furthermore, the different groups contained different numbers of XTH proteins. Group I/II contained the largest number of XTH proteins, including 19 BdXTHs, 18 OsXTHs, 22 AtXTHs, and 27 SIXTHs. Group III was further divided into two subgroups (Group III-A and Group III-B), which included two and six BdXTHs, respectively, along with XTH proteins from the other three species included in the analysis. The Ancestral Group contains the smallest number of XTH proteins: two BdXTHs, one OsXTH, four AtXTHs, and two SIXTHs.

### 2.3. Structural and Conserved Motif Analyses of the BdXTH Genes

**Figure 3:**
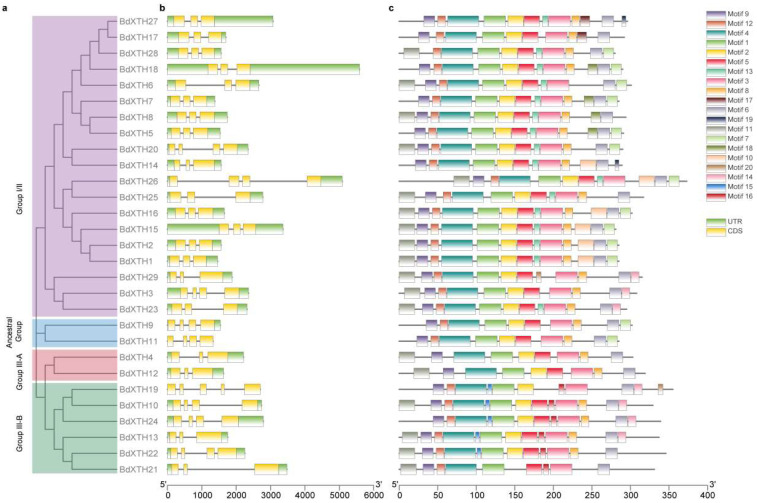
Unrooted neighbor-joining phylogenetic tree, conserved protein motifs, and structural analysis of *BdXTH* genes. (**a**) Evolutionary relationships of the XTH proteins in *Brachypodium*. Proteins from the four clades (Group I/II, Group III-A, Group III-B, and the Ancestral Group) are color coded as in Figure 1. (**b**) The structures of the 29 putative *BdXTH* genes. The UTRs, exons, and introns are represented by green boxes, yellow boxes, and black lines, respectively. (**c**) Conserved motif analysis of the BdXTH proteins. The different motifs are indicated by different colored boxes numbered from motif 1 to motif 20. The structural features of the 20 motifs are shown in Figure S1.

Different combinations of exons and introns can lead to diverse gene functions. To gain more knowledge of the structural diversity of the *BdXTH* genes, the structures of the 29 *BdXTH* genes were analyzed using GSDS. The results showed that the number of exons varied from three to five and that the structures of genes from the same group showed more similarity to one another (Figure 3a,b). Most of the *BdXTH* genes (20/29) contained three exons. Eight genes contained four exons, and only one gene (*BdXTH19*) in Group III-B contained five exons. In addition, only *BdXTH26* from Group I/II contained two longer introns. Moreover, three genes (*BdXTH15*, *18*, and *27*) from Group I/II had longer UTR sequences compared with the other genes.

A conserved motif analysis of all 29 predicted BdXTH protein sequences from *B. distachyon* conducted using the MEME program predicted 20 motifs (Figure 3c and Figure S1); the number of motifs per protein varied from 9 to 13, and members of the same group usually shared a similar motif composition. The proteins in Group III-A and the Ancestral Group had relatively fewer motifs, 9 and 10, respectively, while most of the XTH proteins in the other groups had 12 motifs. Motifs 1, 3, and 4 were found to be highly conserved in all BdXTH proteins. In addition, several conserved motifs were specific to certain groups. For example, motifs 10, 13, and 17–19 were only present in Group I/II proteins, and motifs 15 and 16 were unique to proteins in Group III-B.

### 2.4. Chromosomal Location and Synteny Analysis of the BdXTH Genes

**Figure 4:**
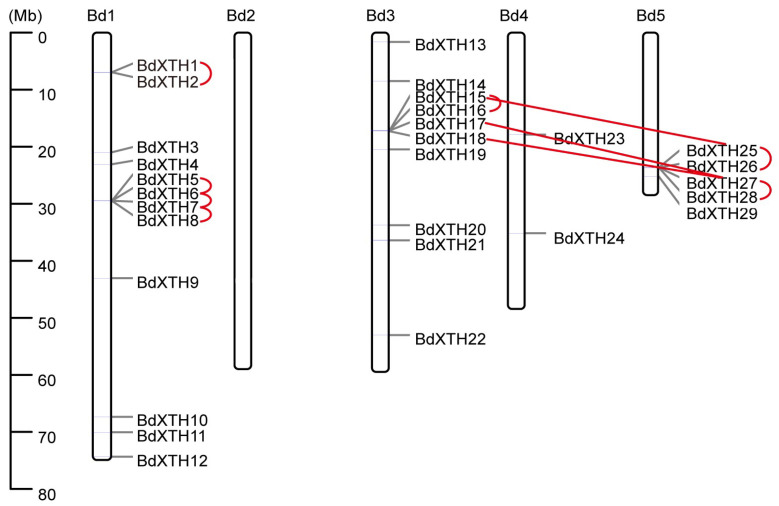
The physical locations of *BdXTH* genes on the five *Brachypodium* chromosomes. Tandemly duplicated gene pairs and segmentally duplicated genes are linked by red lines. The chromosome numbers are displayed at the top of each chromosome, and the scale in megabases (Mb) is shown on the left.

The chromosomal positions of 29 *BdXTH* genes were located using information derived from the *Brachypodium* genome [32]. The *BdXTH* genes were found to be widely distributed on the chromosomes, but the distribution was not uniform (Figure 4). None of the *BdXTH* genes were located on Chr. 2. Only two *BdXTH* genes were located on Chr. 4, and five *BdXTH* genes were located at the end of Chr. 5. In addition, most *BdXTH* genes were located on Chrs. 1 and 3, which had 12 and 10 genes, respectively.

To identify potential duplication events in the *BdXTH* gene family, a collinearity analysis was performed using MCScanX software v1.2. The results revealed that there are three pairs of segmentally duplicated *BdXTH* genes (*BdXTH15/25*, *BdXTH17/27*, and *BdXTH18/27*) and five groups of tandemly duplicated *BdXTH* genes (Figure 4; *BdXTH1/2*, *BdXTH5/6/7/8*, *BdXTH15/16*, *BdXTH25/26*, and *BdXTH27/28*). *BdXTH15*, *BdXTH25*, and *BdXTH27* were involved in both tandem duplications and segmental duplications. Also, all of the duplicated genes are in Group I/II and account for ~48% (14/29) of all *BdXTH* genes, indicating that tandem duplication and segmental duplication have played important roles in the expansion of the *BdXTH* gene family in *B. distachyon*.

### 2.5. Structure-Based Sequence Alignment

**Figure 5:**
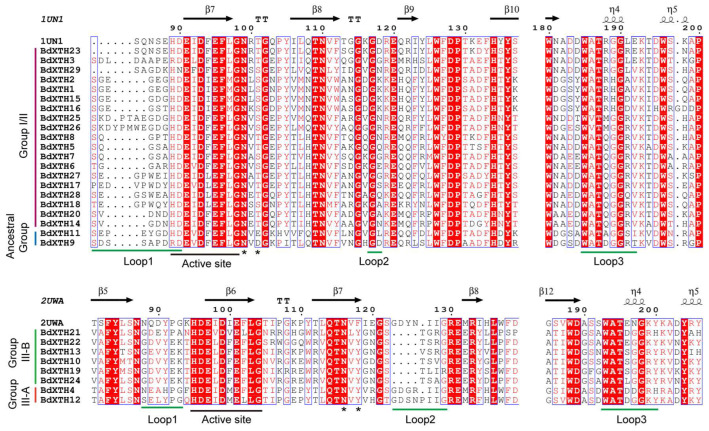
Structure-based sequence alignment of BdXTH proteins. The structures of two proteins (PttXET16-34, PDB id: 1UN1; TmNXG1, PDB id: 2UWA) have been experimentally determined. Proteins in Group I/II and the Ancestral Group had similar structures to 1UN1, and proteins in Group III show similar structures to 2UWA. The active site (ExDxE) and loops 1, 2, and 3 are underlined in black and green, respectively. The *N*-glycosylation site residues are indicated by asterisks. Proteins in Group I/II and the Ancestral Group are shown with purple and blue backgrounds, respectively. Group III is further divided into two subclades, Group III-A and Group III-B, which are indicated with red and green backgrounds, respectively.

The secondary structures of the BdXTH proteins were predicted by aligning the 29 BdXTH protein sequences with those of 2 other proteins for which the structures have been experimentally determined (PttXET16-34, PDB id: 1UN1 and TmNXG1, PDB id: 2UWA) [17,34] using ESPript (http://espript.ibcp.fr/ESPript/cgi-bin/ESPript.cgi (accessed on 26 February 2020)). This analysis showed that the active site (ExDxE) responsible for the catalytic activity [17,34] is highly conserved in all of the BdXTH protein family members (Figure 5). The first glutamate residue (E) is the catalytic nucleophile that initiates the enzymatic reaction, and the second E residue acts as a base to activate the entrant substrate. The *N*-glycosylation site denoted as NxT/S/Y (marked with asterisks) can bind *N*-glycans and is related to protein stability. We found that the *N*-glycosylation site is present in almost all BdXTH proteins except the Ancestral Group members (BdXTH9 and BdXTH11). We also found that the distance between the *N*-glycosylation site and the active site in the Group I/II members is closer than in the Group III members. The BdXTH proteins also contain conserved domains next to the substrate binding site that are called loop 1, loop 2, and loop 3 (underlined in green). Loop 2 in the Group III-A proteins (BdXTH4 and BdXTH12) is longer compared to that in the other groups, which may be the reason why proteins in Group III-A mainly show xyloglucan hydrolase activity.

### 2.6. Cis-Element Analysis of the BdXTH Gene Promoter Regions

**Figure 6:**
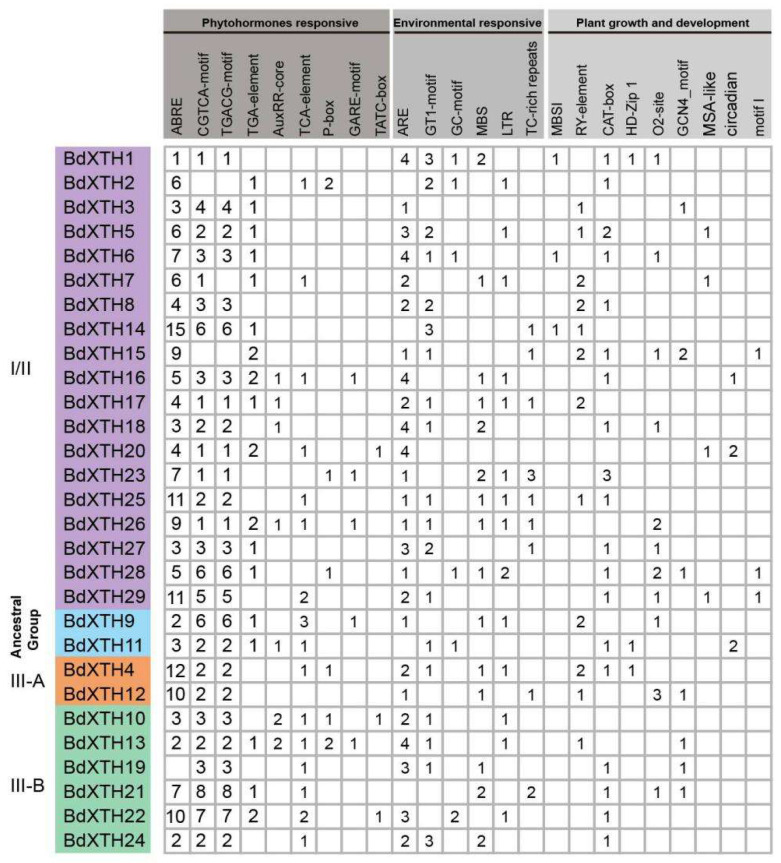
Numbers of *cis*-acting elements in the promoter regions of the 29 *BdXTH* genes. Three types of *cis*-acting elements in the 2000 bp of DNA sequence upstream of the promoter regions are shown in the figure, including phytohormone- and environmentally responsive elements, and plant growth and development-related elements. Members of the different element classes are shown at the top of the figure in different shades of gray. Genes in Group I/II and the Ancestral Group are shown with purple and blue backgrounds, respectively. Group III is further divided into two subclades, Group III-A and Group III-B, which are indicated with orange and green backgrounds, respectively.

XTH family genes play important roles during plant growth and development, as well as in the response to multiple environmental stresses. *Cis*-elements can regulate gene expression via their interactions with trans-acting elements, such as transcription factors. To understand the functions and regulatory network of the *BdXTH* family genes, we analyzed the 2000 bp of DNA sequence upstream of the promoter regions for 29 *BdXTH* genes on the PlantCARE database [35]. As shown in Figure 6, the promoter regions include several hormone-related (abscisic acid, MeJA, auxin, salicylic acid, and gibberellin) *cis*-elements (ABRE, CGTCA-motif, TGACG-motif, TGA-element, AuxRR-core, TCA-element, P-box, GARE-motif, and TATC-box) and growth regulation *cis*-elements (MBSI, RY-element, CAT-box, HD-Zip 1, O2-site, GCN4_motif, MSA-like, and circadian motif I). The promoter regions also contain several environmental response elements, such as those for anaerobic (ARE), drought (MBS), low temperature (LTR), and anoxic (GC-motif) conditions; light-responsive elements (GT1-motif); and defense and stress (TC-rich repeats) *cis*-elements. The distribution of *cis*-elements identified in the promoter regions is shown in Figure S2. *Cis*-acting elements involved in the responses to phytohormones were relatively abundant, especially the abscisic acid-responsive (ABRE) element, and accounted for 27% of the total number of *cis*-elements detected in this study. The *cis*-elements involved in cell cycle regulation (MSA-like), root development (motif I), and flavonoid biosynthesis gene regulation (MBSI) were only found in the promoter regions of several Group I/II genes. In addition, two Group III-B genes (*BdXTH22*, *BdXTH19*) contain the highest (36) and lowest (14) numbers of *cis*-elements identified, respectively.

### 2.7. Expression Profiling of BdXTH Genes in Response to Abiotic Stress and Phytohormone Treatments Using qRT-PCR

**Figure 7:**
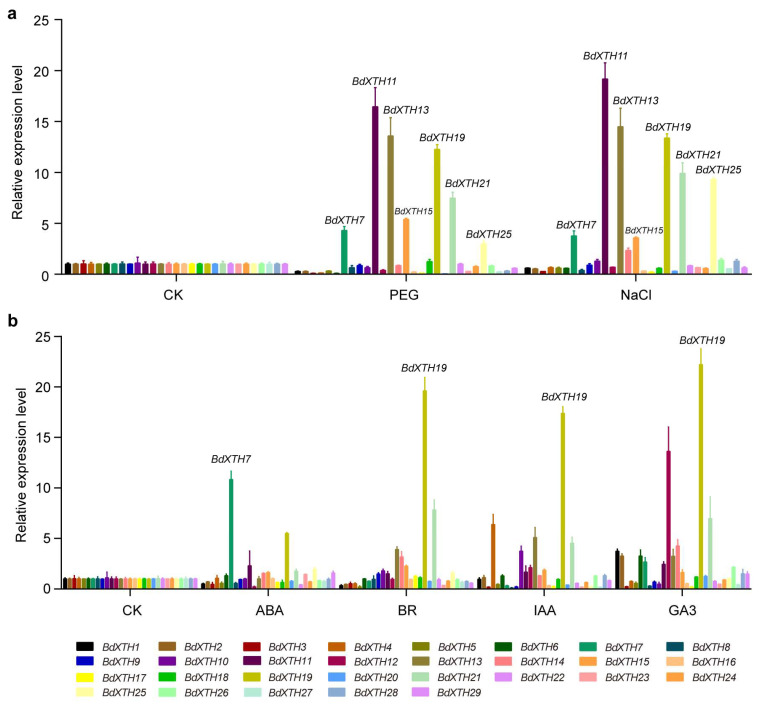
Expression analysis of *BdXTH* genes under different conditions. Quantitative real-time polymerase chain reaction (qRT-PCR) analysis of *BdXTH* gene expression in response to abiotic stresses (drought and salinity) (**a**), and phytohormone treatments (ABA, BR, IAA, and GA3) (**b**). The means ± SD of three biological replicates are present.

Previous studies have shown that the expression of *XTH* family genes can respond to multiple abiotic stresses and phytohormones. To investigate the effect of different treatment conditions on *BdXTH* family genes, qRT-PCR assays were performed to study the expression patterns of 29 *BdXTH* genes in response to drought (PEG), salinity (NaCl), and four plant hormone treatments (ABA, BR, IAA, and GA3) (Figure 7). Analysis of the data showed that the expression levels of many genes were affected by the different treatments. For the PEG and NaCl treatments, the expression patterns of the *BdXTH* genes were similar. Among the up-regulated genes, *BdXTH7/11/13/15/19/21/25* showed relatively higher mRNA levels, in which *BdXTH11* (Ancestral Group) had the highest expression level under both stress treatments. In addition, almost all of the genes that were down-regulated in response to PEG treatment, such as *BdXTH1-6/12/16/17/20/23/27*, also showed relatively lower expression levels under NaCl treatment. For the phytohormone treatments, *BdXTH19* from Group III-B showed the highest mRNA levels in response to BR, IAA, and GA3 treatments, and *BdXTH7* from Group I/II showed the highest level when treated with ABA. The altered expression patterns of the *BdXTH* genes suggest that they might be involved in adaptation to adverse environmental factors or are regulated by diverse plant hormones.

### 2.8. Expression Patterns of the BdXTH Genes

**Figure 8:**
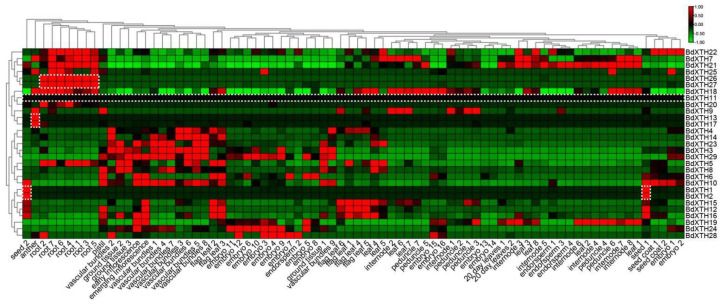
Heat map showing the expression pattern of *BdXTH* genes in *Brachypodium*. Expression profiles from various tissues and developmental stages were downloaded from the NCBI database. The relative expression levels are represented by the colored bars. Red and green boxes indicate high and low expression levels, respectively.

To explore the temporal and spatial expression patterns of the *BdXTH* genes in *Brachypodium*, RNA-seq data, including expression profiles from various tissues and developmental stages, were downloaded from the NCBI database (Table S2). Based on the clusters in the XTH tree in Figure 2, the expression patterns of most *BdXTH* genes exhibited distinct tissue-specific expression patterns (Figure 8). For example, the *BdXTH26/27* genes are mainly expressed in the root, *BdXTH1/2* are mainly expressed in the seed, and *BdXTH13/17* are mainly expressed in the anther, suggesting that the functions of some genes are redundant and that certain genes participate in the developmental progress of specific tissues. In addition, the *BdXTH11* gene showed lower expression levels at almost all developmental stages and in almost all tissues included in the study. Further, we analyzed the expression profiles of the duplicated gene sets, including three segmentally duplicated sets (*BdXTH15/25*, *BdXTH17/27*, and *BdXTH18/27*) and five tandem duplication sets (*BdXTH1/2*, *BdXTH5/6/7/8*, *BdXTH15/16*, *BdXTH25/26*, and *BdXTH27/28*). The result indicated that many of the duplicated gene pairs with close evolutionary relationships, especially the segmentally duplicated sets, showed diversified expression patterns. These results suggest that genes in the *BdXTH* family might have undergone neofunctionalization or sub-functionalization during evolution.

### 2.9. Functional Analysis of the BdXTH27 Gene in Brachypodium

**Figure 9:**
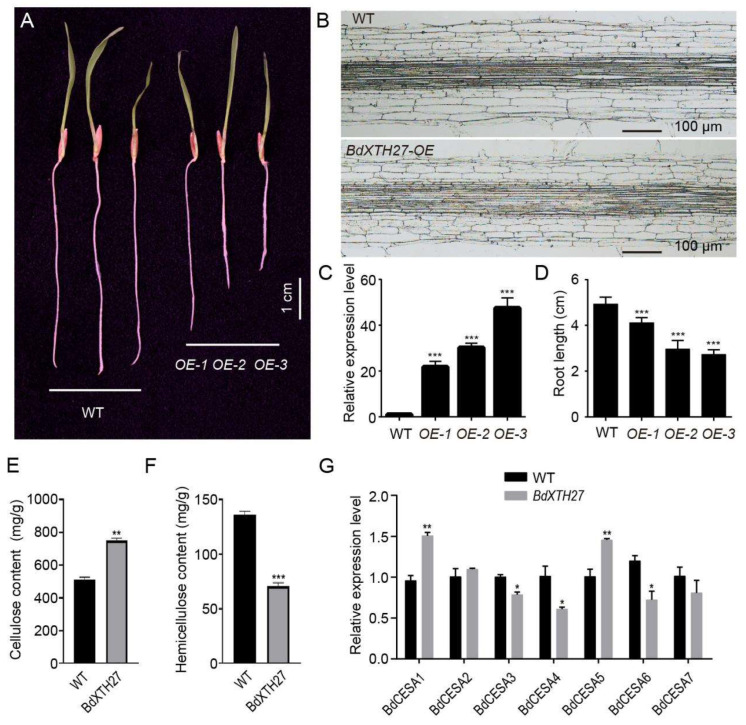
Functional analyses of the *BdXTH27* gene in *B. distachyon*. (**A**) Phenotype of the wild-type (WT) and transgenic *Brachypodium* plants over-expressing the *BdXTH27* gene (OE-1, OE-2, and OE-3), scale bar = 1 cm; (**B**) Root paraffin sections of the *BdXTH27* over-expression and wild-type lines, scale bar = 100 µm; (**C**) Relative expression level of *BdXTH27* gene in roots and (**D**) root length in transgenic lines (OE-1, OE-2, and OE-3) and wild-type (WT). (**E**) The contents of cellulose and (**F**) hemicellulose, and the expression levels of the seven cellulose synthase genes (**G**) of wild-type (WT) and transgenic lines. * *p* < 0.05, ** *p* < 0.01, *** *p* < 0.001.

Analysis of RNA-seq gene expression profile data showed that the *BdXTH27* gene is mainly expressed in the root, which was also confirmed by qRT-PCR (Figure S3). Therefore, we selected the *BdXTH27* gene in order to explore its potential role in root development. Transgenic *BdXTH27* over-expression (OE) lines were obtained by *Agrobacterium*-meditated transformation. Under normal growth conditions, the transgenic lines showed significantly shorter roots than the wild-type plants (Figure 9A), and the root lengths of the OE lines were inversely proportional to the relative expression level of the *BdXTH27* gene (Figure 9C,D). To examine the effect of *BdXTH27* overexpression on cell morphology, we performed paraffin sectioning of roots, and microscopic observation showed that cell lengths in the transgenic lines were significantly shorter than in wild-type roots (Figure 9B). We next analyzed the cell wall components to investigate whether the cell wall components of the transgenic lines had changed. The results showed that the OE lines contained higher levels of cellulose and lower levels of hemicellulose than wild-type plants. (Figure 9E,F). Furthermore, we observed a significant increase in the expression of cellulose synthase genes (*BdCesAs*), such as *BdCesA1* and *BdCesA5*, in the OE lines (Figure 9G).

## 3. Discussion

Xyloglucan endotransglucosylase/hydrolases (XTHs), one family of cell-wall-modifying enzymes, can cut and/or rejoin xyloglucan molecules to regulate the composition and organization of the cell wall [36,37]. In this study, 29 *BdXTH* genes were identified in the *Brachypodium* genome, and an evolutionary analysis showed that they are organized into three major groups (Group I/II, Group III, and the Ancestral Group). A similar XTH protein classification was also reported in wheat [8], *Osmanthus fragrans* [9], and Chinese jujube [11]. The number of *BdXTH* genes in *Brachypodium* was similar to the number of *XTH* genes identified previously in other species, such as *Arabidopsis* (33) [5], rice (29) [6], sugar beet (30) [10], *Chinese jujube* (29) [11], and willow (32) [12], but was much lower than in wheat (135) [8] and banana (53) [15]. Duplication events (tandem duplication or segmental duplications) resulting from whole genome duplication (WGD) via polyploidization or local chromosomal rearrangement played an important role in *XTH* gene family expansion and evolution. Duplicated genes tend to have a close relationship. In our study, all duplicated genes belong to Group I/II, which is the largest group compared to the other groups. For example, the tandemly duplicated gene pairs *BdXTH1/2* and *BdXTH5/6/7/8* on chromosome 1, *BdXTH15/16* on chromosome 3, *BdXTH25/26* and *BdXTH27/28* on chromosome 5, and the segmentally duplicated gene pairs *BdXTH15/25*, *BdXTH17/27*, and *BdXTH18/27* are all members of Group I/II.

The BdXTH proteins in each group contain relatively conserved motifs, and the gene structures are also conserved, suggesting that BdXTH proteins in the same group may perform similar functions (Figure 2 and Figure 4). The results of secondary structure prediction for the translated BdXTH sequences confirm the existence of a highly conserved domain (DEIDFEFLG) that is the catalytic site for both XET and XEH activities [4], especially the three absolutely conserved catalytic residues (ExDxE) in all BdXTH proteins. Also, the *N*-glycosylation site in the BdXTH proteins from Group I/II is adjacent to the catalytic domain, but tends to be located towards the carboxyl terminus in Group III-B proteins. In addition, loop 2 in the Group III-A protein is longer compared to that in the Group III-B members, which has been proposed to be a major structural change responsible for the endo-hydrolase activity of these proteins [17]. Previous studies have reported that XTH proteins in Group III-A mainly display XEH activity [18,19,38], while Group I/II and Group III-B proteins mainly show XET activity [39].

A comparison of the predicted XTH amino acid sequences from *B. distachyon*, rice (*Oryza sativa*), tomato (*Solanum lycopersicum*), and Arabidopsis (*A. thaliana*) revealed a high degree of conservation of the XTH sequences among various plant species, implying a general functional conservation of these proteins in the plant kingdom. On the XTH tree, all clades contained proteins from both the monocot (*Brachypodium* and rice) and dicot (tomato and Arabidopsis) species included in the analysis. This suggests that the precursor genes were present in the most recent common ancestor of monocots and dicots and that closely related proteins might perform similar functions in the different species, although xyloglucan makes up a relatively smaller fraction of the cell wall in Poales species [6,40].

Analysis of the genomic sequences upstream of the *BdXTH* genes using the PlantCARE tool revealed that a series of *cis*-elements involved in the responses to hormones and abiotic stresses, and also in growth and development, are present in the promoter regions, indicating that *BdXTH* genes play diverse roles in *Brachypodium*. Clearly, *BdXTH* gene transcription responds to drought, salinity, and several plant hormones (ABA, BR, IAA, and GA3) based on the changes in gene expression patterns observed in the qRT-PCR experiment (Figure 7), which corroborate the results of the analyses of *BdXTH* gene promoter regions. Expression profiling suggests that *BdXTH* genes might be involved in adaptation to adverse environmental conditions or that they are regulated by many plant hormones. Interestingly, the expression level of *BdXTH19* was up-regulated significantly after two hours of abiotic and hormone stresses in this study, predicting its potential role (Figure 7). In the promoter sequences of the *BdXTH19* gene, *cis*-regulatory elements involved in the MeJA-responsiveness (CGTCA-motif and TGACG-motif), drought-inducibility (MBS), and salicylic acid responsiveness (TCA-element) were identified, which may partly explain why the expression level of *BdXTH19* was consistently up-regulated in most cases examined. But the specific biological functions of the *BdXTH19* gene need to be further studied.

To explore the function of the *BdXTH27* gene, which is mainly expressed in roots, we produced transgenic *BdXTH27*-OE lines, which expressed a short root phenotype. Microscopic examination of paraffin sections indicated that *BdXTH27* may play a role in suppressing the longitudinal extension of roots. In addition, cell wall component analyses showed that the OE plants exhibited higher cellulose levels but lower hemicellulose levels compared to wild-type plants. Furthermore, qRT-PCR data showed that some of the *BdCesA* genes were up-regulated compared with their expression in the wild-type under normal growth conditions. For example, expression of *BdCesA1* and *BdCesA5* was significantly increased (Figure 9g). BdXTH27 belongs to Group I/II, which consists of proteins that mainly show xyloglucan endotransglucosylase activity to elongate xyloglucan chains by cleaving the chains and rejoining the reducing ends to other xyloglucan molecules [39]. Over-expression of *BdXTH27* may modify xyloglucan chains or affect the expression levels of the cellulose synthase genes, affecting the contents of hemicellulose and cellulose in cell walls to regulate root elongation. Characterization of the molecular mechanism will require further study.

## 4. Methods

### 4.1. Identification of the XTH Family Genes in B. distachyon

The latest version of the *Brachypodium distachyon* (v3.1) genome annotation was downloaded from the Phytozome database v12.1.6 (https://phytozome.jgi.doe.gov/pz/portal.html (accessed on 2 January 2020)) [41]. The Hidden Markov Model (HMM) profiles of the XTH protein domains, PF00722 and PF06955, were downloaded from the Pfam database [42] and were used as queries to search the database using the program HMMER3.0 with the default E-value. The online program SMART (http://smart.embl-heidelberg.de/ (accessed on 5 January 2020)) [43] and the PFAM databases (https://pfam.xfam.org (accessed on 5 January 2020)) [44] were used to identify the conserved domains of candidate *Brachypodium* XTH proteins. Only proteins containing both the PF00722 and PF06955 domains were retained for further study. ProtParam (http://web.expasy.org/protparam/ (accessed on 8 January 2020)) was used to predict the physical and chemical features of the BdXTH proteins. The subcellular locations of the BdXTH proteins were predicted using the online website Plant-mPLoc in Cell-PLoc 2.0 (http://www.csbio.sjtu.edu.cn/bioinf/Cell-PLoc-2/ (accessed on 10 January 2020)) [45]. All of the XTH protein sequences from tomato, *Arabidopsis*, and rice were also downloaded from the Phytozome database. The corresponding gene IDs of the XTH protein family members are given in Table S1.

### 4.2. Evolutionary Tree Construction

A phylogenetic tree was constructed using the Neighbor Joining (NJ) method as implemented in MEGA-X software, and the branch support was estimated by bootstrapping with 1000 replicates [46]. Multiple sequence alignments were constructed using ClustalW with default parameters based on 128 amino acid sequences of predicted XTH proteins, of which 29 BdXTHs were from *B. distachyon*, 36 SIXTHs were from *S. lycopersicum*, 30 OsXTHs were from *O. sativa*, and 33 AtXTHs were from *A. thaliana* (Table S1). The evolutionary tree was then visualized using iTol v6.9.1 (https://itol.embl.de/ (accessed on 14 July 2025)) [47].

### 4.3. Gene Structures, Conserved Protein Motifs, and Cis-Acting Regulatory Element Analysis

The *BdXTH* gene structures were displayed using the Gene Structure Display Server (GSDS) tool (http://gsds.cbi.pku.edu.cn/ (accessed on 6 February 2020)) [48] by aligning the cDNA sequences and the corresponding genomic DNA sequences. The Multiple EM for Motif Elicitation (MEME v5.0, http://meme-suite.org/ (accessed on 11 February 2020)) [49] was used to search for possible conserved motifs in the complete amino acid sequences of predicted BdXTH proteins using the default settings. Additionally, the *cis*-elements in the *BdXTH* gene promoter regions (2000-bp of genomic sequence upstream of the coding sequences) were analyzed using the PlantCARE database (http://bioinformatics.psb.ugent.be/webtools/plantcare/html/ (accessed on 16 February 2020)) [35].

### 4.4. Chromosomal Location and Gene Duplication

The chromosomal positions of *BdXTH* genes were acquired from the *Brachypodium distachyon* genome (v 3.1). MCScanX (default parameters) [50] was used to analyze gene duplications using the amino acid sequences and chromosomal location data. The chromosomal locations and gene duplication relationships of the *BdXTH* genes were displayed using TBtools v1.098 (https://github.com/CJ-Chen/TBtools (accessed on 20 February 2020)) [51].

### 4.5. Structure-Based Sequence Alignment Analysis

Alignment of the identified BdXTH protein sequences with two other proteins, TmNXG1 (PDB id: 2UWA) and PttXET16-34 (PDB id: 1UN1), for which the structures have been experimentally determined, was performed to identify common structural elements. The crystal structures of TmNXG1 and PttXET16-34 were obtained from Research Collaboratory for Structural Bioinformatics Protein Data Bank (RCSB PDB, https://www.rcsb.org (accessed on 24 February 2020)). The secondary structures of the BdXTH proteins were then predicted using the online website ESPript (http://espript.ibcp.fr/ESPript/ESPript/ (accessed on 26 February 2020)) [52].

### 4.6. Plant Materials and Treatments

Seeds of *Brachypodium distachyon* (ecotype Bd21) provided by Professor Hailong An of Shandong Agricultural University were placed on wet filter paper and maintained for 3 days at 4 °C in the dark, after which they were cultivated in a plant growth incubator under long-day conditions (18 h light/6 h dark) at 20 °C for one week. The young seedlings were then transferred to custom-made plastic vessels with holes in the bottoms, suspended on the surface of a reservoir containing 0.5× MS liquid medium, and maintained in a growth chamber under the same conditions (18 h light/6 h dark, 20 °C). Seedlings with three leaves were subjected to different abiotic stress and phytohormone treatments, which included 150 mM NaCl, 20% polyethylene glycol (PEG) 6000, 1 µM 3-indole acetic acid (IAA), 1 µM gibberellic acid (GA_3_), 1 µM abscisic acid (ABA), and 1 µM 24-epibrassinolide (BR). Roots of the stress-treated and control plants were collected after 2 h of treatment. All of the materials were immediately frozen in liquid nitrogen and stored at −80 °C prior to RNA extraction. Every sample consisted of three independent biological replications.

### 4.7. Expression Pattern Analysis of BdXTH Genes Using the Public RNA-Seq Data

The publicly available RNA-seq data from 68 *Brachypodium* tissue samples (Table S2) were downloaded from the NCBI database (https://www.ncbi.nlm.nih.gov/ (accessed on 1 March 2020)) using the SRP series accession numbers (SRP008505 [53], SRP295302, SRP309091, and SRP295028-SRP295071). TBtools (https://github.com/CJ-Chen/TBtools (accessed on 21 March 2020)) [51] was used to create the heat maps and hierarchical clustering using FPKM values extracted from the RNA-seq dataset.

### 4.8. RNA Isolation and qRT-PCR Gene Expression Analysis

The frozen samples were ground into powder in liquid nitrogen with a mortar and pestle. Total RNA was isolated from roots using the RNAprep Pure Plant Kit (Tiangen, Beijing, China), and first-strand total cDNA was then synthesized using the HiScript^®^ II Q RT SuperMix for qPCR (+gDNA wiper) Kit (Vazyme, Nanjing, China). Beacon designer software (Premier Biosoft, Palo Alto, CA, USA) was used to design the gene-specific primers for qRT-PCT (Table S3). Real-time qRT-PCR assays were performed using ChamQ^TM^ SYBR^®^ qPCR Master Mix (Vazyme, China) on a Bio-Rad CFX96 Real-time PCR System (Bio-Rad, Hercules, CA, USA). The *BdUBC18* gene was used as the internal control for normalization of gene expression. Each PCR contained 0.4 μL of each primer, 1 μL of template cDNA, and 10 μL of 2XChamQ SYBR qPCR Master Mix in a final volume of 20 μL. The thermal cycling protocol was as follows: 95 °C for 30 s, followed by 40 cycles of 95 °C for 10 s and 60 °C for 30 s. Subsequently, melting curves were performed to confirm the specificity of the primers. Each reaction was performed three times, and the 2^−ΔΔCt^ method [54] was used to calculate the relative gene expression levels.

### 4.9. Identification of the BdCESA Genes in Brachypodium

The *BdCESA* genes were identified by comparisons with the homologous *CESA* genes from rice and Arabidopsis, and the conserved domains of the candidate genes were further verified using the online program SMART (http://smart.embl-heidelberg.de/ (accessed on 24 March 2022)) [43] and the PFAM databases (https://pfam.xfam.org (accessed on 24 March 2022)) [44]. Only genes containing both the PF13632 and PF14569 domains were considered to be *BdCESA* genes. A total of 7 *BdCESA* genes were identified in *Brachypodium distachyon* (Table S4).

### 4.10. Analysis of the BdXTH27-OE Transgenic Lines

Seeds of transgenic plants over-expressing *BdXTH27* and the wild-type were placed on wet filter paper and maintained for 3 days at 4 °C in the dark, after which they were cultivated in a plant growth incubator under long-day conditions (18 h light/6 h dark) at 20 °C for 5 days. The root lengths were then measured, and paraffin sectioning was used to further observe the morphology of the root tip cells [55]. The young seedlings were then transferred to custom-made plastic vessels with holes in the bottoms, suspended on the surface of a reservoir containing 0.5× MS liquid medium, and maintained in a growth chamber under the same conditions (18 h light/6 h dark, 20 °C). After three weeks of continuous growth, seedlings were used to measure the cellulose content of plant cell walls by the anthrone assay method [56] and also for the extraction of RNA to check the expression levels of the cellulose synthase genes.

## 5. Conclusions

In this study, we performed a genome-wide analysis of the *BdXTH* gene family in *Brachypodium distachyon* and investigated the expression profiles of all 29 genes in different tissues and at different developmental stages, as well as in response to various stress conditions. Changes in the relative expression of most *BdXTH* genes under diverse abiotic stresses suggest their possible functions in drought, salinity, and responses to several phytohormones. In addition, many of the *BdXTH* genes exhibit distinct tissue-specific expression patterns. When the *BdXTH27* gene, which is mainly expressed in roots, was over-expressed in *Brachypodium*, root length decreased in the transgenic plants that exhibited higher cellulose levels but lower hemicellulose levels compared to wild-type plants. These results may advance our understanding of the role of *BdXTH* genes in the regulation of *Brachypodium* growth and development and in its response to abiotic stresses.

## Figures and Tables

**Table 1:** Molecular characterization of *BdXTH* genes.

Gene Name	Gene ID	Chromosome	Start	End	Length (aa)	MW (kDa)	PI	II	AI	GRAVY	Subcellular Localization
BdXTH1	Bradi1g09690	Bd1	6944672	6946132	284	31.55	5.67	36.99	63.87	−0.27	Cell wall
BdXTH2	Bradi1g09700	Bd1	6956014	6957576	284	31.54	5.67	36.12	63.87	−0.26	Cell wall
BdXTH3	Bradi1g25847	Bd1	20978556	20980916	307	34.16	5.09	39.79	63.03	−0.34	Cell wall
BdXTH4	Bradi1g27867	Bd1	23074430	23076645	302	34.11	8.73	40.95	58.91	−0.46	Cell wall
BdXTH5	Bradi1g33810	Bd1	29475206	29476739	290	32.39	8.78	30.99	58.24	−0.42	Cell wall
BdXTH6	Bradi1g33817	Bd1	29478854	29481513	300	34.28	6.71	43.09	71.2	−0.36	Cell wall
BdXTH7	Bradi1g33827	Bd1	29485795	29487172	284	31.45	5.73	38.54	62.64	−0.34	Cell wall
BdXTH8	Bradi1g33840	Bd1	29494938	29496678	293	32.59	6.22	31.26	69.66	−0.28	Cell wall/Cytoplasm
BdXTH9	Bradi1g44777	Bd1	43043568	43045106	301	33.45	5.05	43.37	68.37	−0.33	Cell wall
BdXTH10	Bradi1g68590	Bd1	67356916	67359656	328	36.46	6.02	52.51	67.65	−0.39	Cell wall
BdXTH11	Bradi1g71937	Bd1	70046381	70048307	284	31.51	6.44	36.9	78.27	−0.12	Cell wall
BdXTH12	Bradi1g77990	Bd1	74331199	74332831	318	35.24	7.00	46.49	61.07	−0.41	Cell wall
BdXTH13	Bradi3g02700	Bd3	1633396	1635150	336	37.66	8.57	51.44	76.10	−0.22	Cell wall
BdXTH14	Bradi3g10290	Bd3	8496465	8498025	288	32.10	4.91	32.13	66.32	−0.33	Cell wall
BdXTH15	Bradi3g18590	Bd3	17168440	17171803	280	31.02	4.91	38.97	59.21	−0.38	Cell wall
BdXTH16	Bradi3g18600	Bd3	17175111	17176766	301	33.71	5.29	28.49	60.66	−0.52	Cell wall
BdXTH17	Bradi3g18607	Bd3	17180627	17182323	291	33.41	4.85	42.45	63.68	−0.51	Cell wall
BdXTH18	Bradi3g18690	Bd3	17286604	17292191	289	32.83	6.89	32.97	71.87	−0.41	Cell wall
BdXTH19	Bradi3g21337	Bd3	20473847	20476548	354	39.32	8.75	48.62	75.06	−0.25	Cell wall
BdXTH20	Bradi3g31767	Bd3	20473847	20476548	289	32.32	5.78	26.27	69.24	−0.26	Cell wall
BdXTH21	Bradi3g34227	Bd3	36405786	36409262	330	35.58	7.83	44.98	79.88	−0.18	Cell wall
BdXTH22	Bradi3g52307	Bd3	53017512	53019765	345	37.56	8.83	33.85	64.09	−0.23	Cell wall
BdXTH23	Bradi4g16990	Bd4	17884860	17887177	294	33.37	6.25	37.64	64.76	−0.49	Cell wall/Cytoplasm
BdXTH24	Bradi4g29707	Bd4	35169644	35172434	338	37.31	6.41	52.96	70.74	−0.29	Cell wall
BdXTH25	Bradi5g20718	Bd5	23655071	23657848	316	34.23	4.67	44.62	44.62	−0.19	Cell wall
BdXTH26	Bradi5g20726	Bd5	23658483	23663566	372	40.94	5.79	43.33	67.96	−0.25	Cell wall
BdXTH27	Bradi5g20734	Bd5	23661776	23664848	295	33.66	4.83	38.07	76.41	−0.29	Cell wall
BdXTH28	Bradi5g20742	Bd5	23668827	23670387	279	30.54	5.19	40.05	67.89	−0.26	Cell wall
BdXTH29	Bradi5g22907	Bd5	25246820	25248701	314	34.27	5.37	31.8	73.34	−0.25	Cell wall

## Data Availability

All of the data and materials supporting our research findings are contained in the Section 4. Details are provided in the attached additional files.

## Supplementary Materials

The following supporting information can be downloaded at: https://www.mdpi.com/article/10.3390/ijms26157457/s1.

## Abbreviations

XTH: xyloglucan endotransglucosylase/hydrolase; IAA: auxin; GA3: gibberellic acid; ABA: abscisic acid; BR: brassinolide; MW: molecular weight; MeJA: methyl jasmonate; PEG: polyethylene glycol; qRT-PCR: quantitative real-time polymerase chain reaction; GSDS: Gene Structure Display Server; NJ: neighbor-joining.
