# Supplementary material for: Genome-Wide Analyses of the XTH Gene Family in Brachypodium distachyon and Functional Analyses of the Role of BdXTH27 in Root Elongation
Source: Int J Mol Sci. 2025 Aug 1;26(15):7457. doi: 10.3390/ijms26157457 (PMC12347618; doi:10.3390/ijms26157457)
Supplement: Supplementary file 1 [file ijms-26-07457-s001.zip › ijms-3734577-supplementary.pdf]

Table S1 Identification of XTH gene family members from three plant species.

Table S2 The publicly available RNA-seq data from different tissues and developmental stages throughout the life cycles of *Brachypodium distachyon.*

Table S3 Oligonucleotide primers used for qRT-PCR assays in this study.

Table S4 Information of the seven cellulose synthase genes in *Brachypodium distachyon* .

Figure S1 Sequence logos of 20 conserved motifs detected in the BdXTH proteins. The overall height of each stack represents the degree of conservation at that position, and the height of the individual letter within each stack indicates the relative frequency of the corresponding amino acid.

Figure S2 *Cis*-acting elements identified in the 2,000 bp of sequence upstream of the promoter regions of the *BdXTH* genes.

Figure S3 Expression analysis of the *BdXTH27* gene in different tissue samples by real-time fluorescence quantitative PCR.

Table S1 Identification of XTH gene family members from three plant species.

Species	Name	Gene ID
	OsXTH1	LOC_Os02g03550
	OsXTH2	LOC_Os02g17880
	OsXTH3	LOC_Os02g17900
	OsXTH4	LOC_Os02g46910
	OsXTH5	LOC_Os02g57770
	OsXTH6	LOC_Os03g01800
	OsXTH7	LOC_Os03g13570
	OsXTH8	LOC_Os03g63760
	OsXTH9	LOC_Os04g51450
	OsXTH10	LOC_Os04g51460
	OsXTH11	LOC_Os04g51510
	OsXTH12	LOC_Os04g51520
	OsXTH13	LOC_Os04g53950
	OsXTH14	LOC_Os06g13040
	OsXTH15	LOC_Os06g22919
	OsXTH16	LOC_Os06g48160
	OsXTH17	LOC_Os06g48170
	OsXTH18	LOC_Os06g48180
	OsXTH19	LOC_Os06g48200
	OsXTH20	LOC_Os07g29750
	OsXTH21	LOC_Os07g34580
	OsXTH22	LOC_Os08g13920
	OsXTH23	LOC_Os08g13980
	OsXTH24	LOC_Os08g14200
	OsXTH25	LOC_Os08g14210
	OsXTH26	LOC_Os09g23220
	OsXTH27	LOC_Os10g02770
	OsXTH28	LOC_Os10g39840
	OsXTH29	LOC_Os10g42670
rice	OsXTH30	LOC_Os11g33270
	SIXTH1	Solyc01g005120
	SIXTH2	Solyc01g081060
	SIXTH3	Solyc01g099630
	SIXTH4	Solyc01g106650
	SIXTH5	Solyc02g080160
	SIXTH6	Solyc02g091920
	SIXTH7	Solyc03g031800

	SlXTH8	Solyc03g093080
	SlXTH9	Solyc03g093110
	SlXTH10	Solyc03g093120
	SlXTH11	Solyc03g093130
	SlXTH12	Solyc03g098430
	SlXTH13	Solyc04g008210
	SlXTH14	Solyc05g005680
	SlXTH15	Solyc05g046290
	SlXTH16	Solyc05g053700
	SlXTH17	Solyc06g083400
	SlXTH18	Solyc07g006850
	SlXTH19	Solyc07g006860
	SlXTH20	Solyc07g006870
	SlXTH21	Solyc07g009380
	SlXTH22	Solyc07g052980
	SlXTH23	Solyc07g055990
	SlXTH24	Solyc07g056000
	SlXTH25	Solyc08g076080
	SlXTH26	Solyc09g008320
	SlXTH27	Solyc09g092520
	SlXTH28	Solyc10g005350
	SlXTH29	Solyc11g040140
	SlXTH30	Solyc11g065600
	SlXTH31	Solyc11g066270
	SlXTH32	Solyc12g007250
	SlXTH33	Solyc12g007260
	SlXTH34	Solyc12g007270
	SlXTH35	Solyc12g017240
tomato	SlXTH36	Solyc12g098610
	AtXTH1	AT4G13080
	AtXTH2	AT4G13090
	AtXTH3	AT3G25050
	AtXTH4	AT2G06850
	AtXTH5	AT5G13870
	AtXTH6	AT5G65730
	AtXTH7	AT4G37800
	AtXTH8	AT1G11545
	AtXTH9	AT4G03210
	AtXTH10	AT2G14620
	AtXTH11	AT3G48580
	AtXTH12	AT5G57530
	AtXTH13	AT5G57540

	AtXTH14	AT4G25820
	AtXTH15	AT4G14130
	AtXTH16	AT3G23730
	AtXTH17	AT1G65310
	AtXTH18	AT4G30280
	AtXTH19	AT4G30290
	AtXTH20	AT5G48070
	AtXTH21	AT2G18800
	AtXTH22	AT5G57560
	AtXTH23	AT4G25810
	AtXTH24	AT4G30270
	AtXTH25	AT5G57550
	AtXTH26	AT4G28850
	AtXTH27	AT2G01850
	AtXTH28	AT1G14720
	AtXTH29	AT4G18990
Arabidopsis	AtXTH30	AT1G32170
	AtXTH31	AT3G44990
	AtXTH32	AT2G36870
	AtXTH33	AT1G10550

Table S2 The publicly available RNA-seq data from different tissues and developmental stages throughout the life cycles of *Brachypodium distachyon*.

Accession number	Tissues or organ	Abbreviation	Developmental stages
SRP008505	leaves	20 day leaves 1	20 Day Leaves
	leaves	20 day leaves 2	20 Day Leaves
	Inflorescence	early inflorescence	early Inflorescence
	Inflorescence	emerging inflorescence	emerging Inflorescence
	Pistil	pistil	pistil
	Embryo	embryo 15	embryo 25 Days After pollination
	25 Days After Pollination	embryo 16	embryo 25 Days After pollination
	Anther	anther	anther
	Seed	seed 1	seed (5 Days after pollination)
	Seed	seed 2	seed (10 Days after pollination)
SRP295302	ground tissue（Control_1 ）	ground tissue 1	19day
	ground tissue（Control_2 ）	ground tissue 2	19day
	ground tissue（Control_3 ）	ground tissue 3	19day
	meristem（vascular bundles stages Stage1_1）	vascular bundles 1	19day no perceptible protoxylem cell
	meristem （vascular bundles stages Stage1_2）	vascular bundles 2	19day no perceptible protoxylem cell
	meristem （vascular bundles stages Stage1_3）	vascular bundles 3	19day no perceptible protoxylem cell
	meristem （vascular bundles stages Stage2_1）	vascular bundles 4	19day 1 to 2 cells of protoxylem cells
	meristem （vascular bundles stages Stage2_2）	vascular bundles 5	19day 1 to 2 cells of protoxylem cells
	meristem （vascular bundles stages Stage2_3）	vascular bundles 6	19day 1 to 2 cells of protoxylem cells
	meristem （vascular bundles stages Stage3_1）	vascular bundles 7	19day 3 to 4 cells of protoxylem cells
	meristem （vascular bundles stages Stage3_2）	vascular bundles 8	19day 3 to 4 cells of protoxylem cells
	meristem （vascular bundles stages Stage3_3）	vascular bundles 9	19day 3 to 4 cells of protoxylem cells
	embryo	embryo 1	2 cell and quadrant stage embryo

	embryo	embryo 2	2 cell and quadrant stage embryo
	embryo	embryo 3	leaf early stage embryo
	embryo	embryo 4	leaf early stage embryo
	embryo	embryo 5	leaf middle stage embryo
	embryo	embryo 6	leaf middle stage embryo
	embryo	embryo 7	leaf late stage embryo
	embryo	embryo 8	leaf late stage embryo
	embryo	embryo 9	preembryo stage embryo
SRP309091	embryo	embryo 10	preembryo stage embryo
	embryo	embryo 11	transition stage embryo
	embryo	embryo 12	transition stage embryo
	embryo	embryo 13	mature stage embryo
	embryo	embryo 14	mature stage embryo
	endorsperm	endorsperm 1	early endorsperm stage
	endorsperm	endorsperm 2	early endorsperm
	endorsperm	endorsperm 3	late endorsperm stage
	endorsperm	endorsperm 4	late endorsperm
	seed coat	seed coat 1	seed coat
	seed coat	seed coat 2	seed coat
	flag leaf	flag leaf 1	flag leaf division
	flag leaf	flag leaf 2	flag leaf division
	flag leaf	flag leaf 3	flag leaf elongation
	flag leaf	flag leaf 4	flag leaf elongation
	flag leaf	flag leaf 5	flag leaf maturation
	flag leaf	flag leaf 6	flag leaf maturation
	internode	internode 1	internode division zone
	internode	internode 2	internode division zone
	internode	internode 3	internode division zone
	internode	internode 4	internode elongation zone
	internode	internode 5	internode elongation zone
	internode	internode 6	internode elongation zone
	internode	internode 7	internode maturation zone
	internode	internode 8	internode maturation zone
	flag leaf	leaf 1	leaf division zone
	leaf	leaf 2	leaf division zone
	leaf	leaf 3	Leaf division zone
	leaf	leaf 4	leaf elongation zone
SRP295028-SRP295071	leaf	leaf 5	leaf elongation zone
	leaf	leaf 6	leaf maturation zone
	leaf	leaf 7	leaf maturation zone
	leaf	leaf 8	leaf maturation zone
	peduncle	peduncle 1	peduncle division

peduncle	peduncle 2	peduncle division
peduncle	peduncle 3	peduncle division
peduncle	peduncle 4	peduncle elongation
peduncle	peduncle 5	peduncle elongation
peduncle	peduncle 6	peduncle maturation
peduncle	peduncle 7	peduncle maturation
Root	root 1	Root division zone
Root	root 2	Root division zone
Root	root 3	Root division zone
Root	root 4	Root elongation zone
Root	root 5	Root elongation zone
Root	root 6	Root elongation zone
Root	root 7	Root maturation zone

Table S3 Oligonucleotide primers used for qRT-PCR assays in this study.

Primer name	Sequence
Bradi1g09690.1F	CCGAGGTTTACCTGCGTTCT
Bradi1g09690.1R	CCTCTCCGTAAGACTGTCGC
Bradi1g09700.1F	CCGAGGTTTACCTGCGTTCT
Bradi1g09700.1R	CCTCTCCGTAAGACTGTCGC
Bradi1g25847.1F	GCGGTGTAACTATCAGAGA
Bradi1g25847.1R	CTACCCATGCTCGTGTAT
Bradi1g27867.1F	TTCGTTTCACAAGCATAGC
Bradi1g27867.1R	AGTAGCATTGAGAACATCCA
Bradi1g33810.1F	AGCTCAGCGACATGAGCTAC
Bradi1g33810.1R	AGTTTCTCTCAACGGCGGAG
Bradi1g33817.1F	CGTGGCAGTGTAAGAATC
Bradi1g33817.1R	AAACGAGTGGAGAAACCT
Bradi1g33827.1F	GAAGCCCGTGGATGTACCAG
Bradi1g33827.1R	GTGTCGGCGCAGTAGTTGTA
Bradi1g33840.1F	ATGCCTCGCTCATTCCACTC
Bradi1g33840.1R	TCGCTATTATCGCCGACACC
Bradi1g44777.1F	CCAGACCAACGTCTTCGTCA
Bradi1g44777.1R	CCTGTAGTCGTGGAAGTCGG
Bradi1g68590.1F	AAGGAAGGAACGGAACGGAC
Bradi1g68590.1R	GCTGCTGCTTCGCTAAACTC
Bradi1g71937.1F	GAGGATGAAGATACCCGGCG
Bradi1g71937.1R	AAAACGACGTGCTTGCCTTC
Bradi1g77990.1F	ACGGATCGCTCCAAGAATCG
Bradi1g77990.1R	CGTTAATCAATCGCCGCTGG
Bradi3g02700.1F	TGAGTTCCTCGGCAACATCC
Bradi3g02700.1R	GTCCACAGGATGGAGTAGCG
Bradi3g10290.1F	ATCCGGCCAGTTAAGCGAAA

Bradi3g10290.1R	GCCGGACACAAGTCCGATAA
Bradi3g18590.1F	GGCAGTTGGCACGTTAGTTG
Bradi3g18590.1R	CATCCAAATCGCCTCCCCAT
Bradi3g18600.1F	TTTTGCCACCGTCGTTGTTC
Bradi3g18600.1R	TCCTTGACACAGCAGCTACG
Bradi3g18607.1F	GCGCGAGACAAGCACATGAT
Bradi3g18607.1R	CAGTCCGAGGTGCATTCCTT
Bradi3g18690.1F	AGTGAACAACGGGACAACCA
Bradi3g18690.1R	CAGAACACCTACGGCCTCAC
Bradi3g21337.2F	GGTTACTGCTACGACCGTCT
Bradi3g21337.2R	CTGATCCCTGGACCTCGCTA
Bradi3g31767.1F	GTCCGATGTACGGGTTCTCC
Bradi3g31767.1R	GGCCTTGCTCCAATCAGTCT
Bradi3g34227.1F	TACCGCCATGGCTTCTTCAG
Bradi3g34227.1R	GACAGGTAGAAGGCGACGAC
Bradi3g52307.1F	GACGCCGGATGATAGGACTG
Bradi3g52307.1R	GCCGTGGAGGTAGTAATCCG
Bradi4g16990.1F	CGCGCCCAATTAATCCAGTG
Bradi4g16990.1R	TACGTACGCCAATCGACCAG
Bradi4g29707.1F	GATGTGATCCCGCCATGACT
Bradi4g29707.1R	AAATTCCGACCGGTCTCCAC
Bradi5g20718.1F	GAGTTCCTGGGGAACGTCAG
Bradi5g20718.1R	AACCACAGGTAGAACCGCTG
Bradi5g20726.1F	TTAACGCGTGCAGAGGCTAT
Bradi5g20726.1R	GGACAGACACACAGAGCGAA
Bradi5g20734.1F	CAAACGAGGTTCTCCCTCCC
Bradi5g20734.1R	AAATGTGATATGCGCGTGGC
Bradi5g20742.1F	AAGTCAAGACGGACTGGTCG

Bradi5g20742.1R	CACGTACTCCTGCCCGTATC
Bradi5g22907.1F	GGCGTCGTCACTGCTTTCTA
Bradi5g22907.1R	GTACAGGTTCGTCTGCACCA
BdUBC18-F	TGGAGGCACCTCAGGTCATTTTC
BdUBC18-R	GTTGCTTTGCTGGCGAGCTAGAC

Table S4 Information of the seven cellulose synthase genes in *Brachypodium distachyon* .

Name	locusName	Pfam	Best-hit-arabi-name	arabi-symbol	arabi-defline	Best-hit-rice-name	rice-defline
BdCESA1	Bradi1g02510	PF13632,PF14569	AT5G64740.1	CESA6	cellulose synthase 6	LOC_Os03g62090.1	CESA5 - cellulose synthase, expressed
BdCESA2	Bradi1g04597	PF13632,PF14569	AT5G05170.1	ATCESA3	Cellulose synthase family protein	LOC_Os03g59340.1	CESA2 - cellulose synthase, expressed
BdCESA3	Bradi1g53207	PF13632,PF14569	AT5G64740.1	CESA6	cellulose synthase 6	LOC_Os07g14850.1	CESA6 - cellulose synthase, expressed
BdCESA4	Bradi1g54250	PF13632,PF14569	AT5G05170.1	ATCESA3	Cellulose synthase family protein	LOC_Os07g10770.1	CESA8 - cellulose synthase, expressed
BdCESA5	Bradi2g34240	PF13632,PF14569	AT4G32410.1	AtCESA1	cellulose synthase 1	LOC_Os05g08370.1	CESA1 - cellulose synthase, expressed
BdCESA6	Bradi3g28350	PF13632,PF14569	AT5G44030.1	CESA4	cellulose synthase A4	LOC_Os10g32980.1	CESA7 - cellulose synthase, expressed
BdCESA7	Bradi4g30540	PF13632,PF14569	AT5G17420.1	ATCESA7	Cellulose synthase family protein	LOC_Os09g25490.1	CESA9 - cellulose synthase, expressed

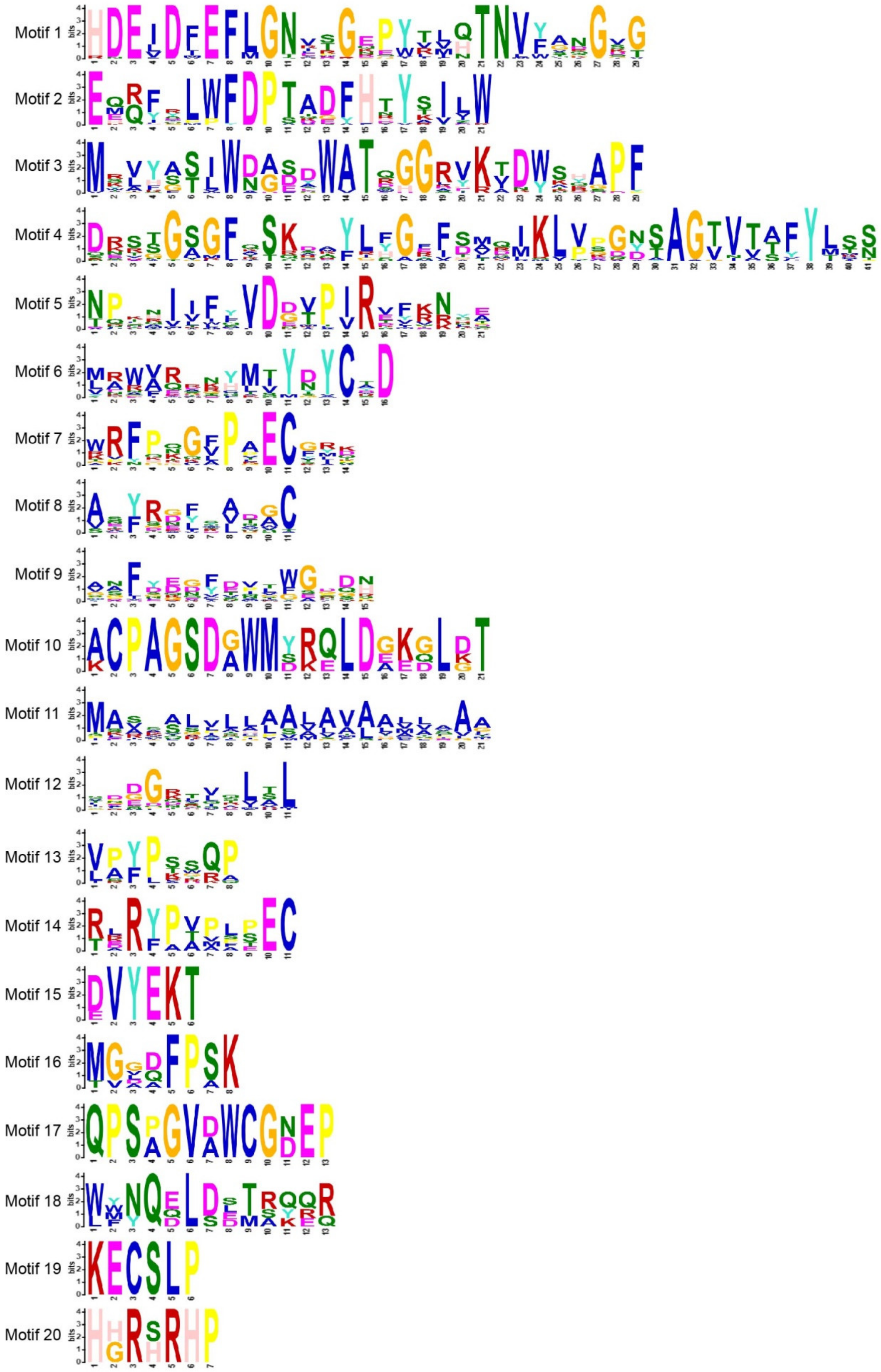

Figure S1 Sequence logos of 20 conserved motifs detected in the BdXTH proteins. The overall height of each stack represents the degree of conservation at that position, and the height of the individual letter within each stack indicates the relative frequency of the corresponding amino acid.

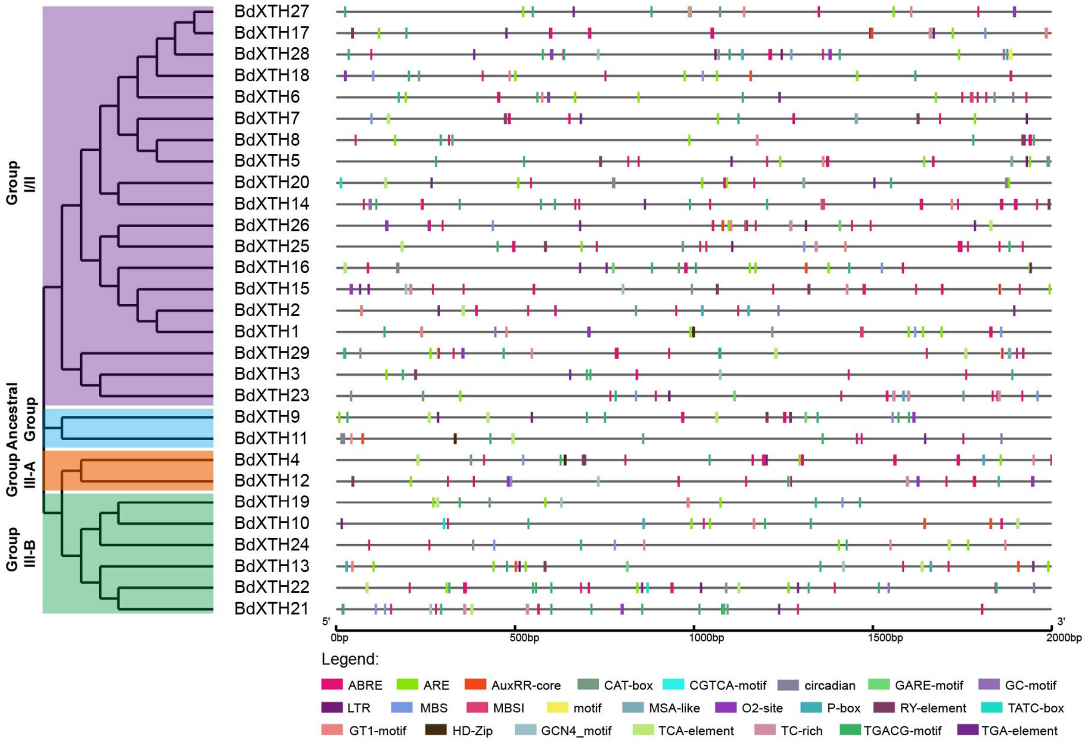

Figure S2 *Cis*-acting elements identified in the 2,000 bp of sequence upstream of the promoter regions of the *BdXTH* genes.

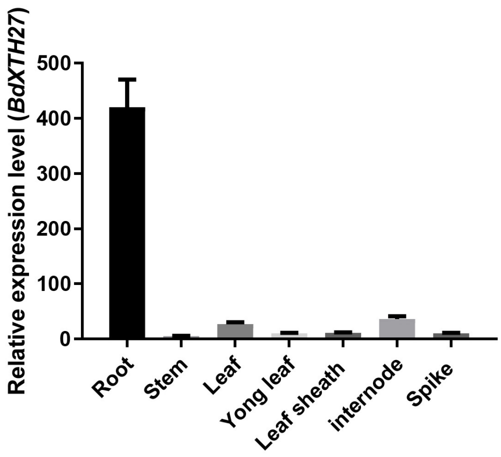

Figure S3 Expression analysis of the *BdXTH27* gene in different tissue samples by real-time fluorescence quantitative PCR.
